# Influence of Manufacturing Methods of Implant-Supported Crowns on External and Internal Marginal Fit: A Micro-CT Analysis

**DOI:** 10.1155/2018/5049605

**Published:** 2018-01-23

**Authors:** Izabela C. M. Moris, Silas Borges Monteiro, Raíssa Martins, Ricardo Faria Ribeiro, Erica A. Gomes

**Affiliations:** ^1^School of Dentistry, University of Ribeirão Preto, Ribeirão Preto, SP, Brazil; ^2^Department of Dental Materials and Prosthodontics, School of Dentistry of Ribeirão Preto, University of São Paulo, Ribeirão Preto, SP, Brazil

## Abstract

**Aim:**

To evaluate the influence of different manufacturing methods of single implant-supported metallic crowns on the internal and external marginal fit through computed microtomography.

**Methods:**

Forty external hexagon implants were divided into 4 groups (*n* = 8), according to the manufacturing method: GC, conventional casting; GI, induction casting; GP, plasma casting; and GCAD, CAD/CAM machining. The crowns were attached to the implants with insertion torque of 30 N·cm. The external (vertical and horizontal) marginal fit and internal fit were assessed through computed microtomography. Internal and external marginal fit data (*μ*m) were submitted to a one-way ANOVA and Tukey's test (*α* = .05). Qualitative evaluation of the images was conducted by using micro-CT.

**Results:**

The statistical analysis revealed no significant difference between the groups for vertical misfit (*P* = 0.721). There was no significant difference (*P* > 0.05) for the internal and horizontal marginal misfit in the groups GC, GI, and GP, but it was found for the group GCAD (*P* ≤ 0.05). Qualitative analysis revealed that most of the samples of cast groups exhibited crowns underextension while the group GCAD showed overextension.

**Conclusions:**

The manufacturing method of the crowns influenced the accuracy of marginal fit between the prosthesis and implant. The best results were found for the crowns fabricated through CAD/CAM machining.

## 1. Introduction

Despite the increasing indication of ceramic prostheses due to esthetic requirements, the ceramic-fused-to-metal prostheses remain as the gold standard in oral rehabilitation, as a result of higher predictability in addition to satisfactory mechanical properties and clinical performance [1, 2]. However, factors such as composition, casting technique, and alloy injection must be previously evaluated in order to enhance the long-term success of ceramic-fused-to-metal prostheses [3].

The base-metal alloys have been used for fabrication of ceramic-fused-to-metal prostheses because of lower cost in comparison to noble metal alloys, biocompatibility, and satisfactory clinical performance. On the other hand, those alloys are more sensitive to casting technique as a result of high melting point and oxidation, which can reduce the accuracy of restorations [3].

The heating sources as torch, induction, and electric arch are commonly used for melting of metallic alloys [3], and the lost-wax casting technique (conventional method) associated with centrifugation for alloy injection is widely used in dentistry [4]. However, the conventional method presents some limitations on control of alloy temperature and changes in composition as a result of overheating [5]. Thus, other techniques (e.g., induction casting and plasma casting) have been developed in order to achieve the ideal control of melting temperature for getting a homogeneous bulk including all alloy components. Recently, the Computer Aided Design/Computer Aided Manufacturing (CAD/CAM) system has been also indicated for fabrication of implant-supported metallic frameworks [3, 6, 7] in order to get adapted and satisfactory prosthetic restorations.

A key point for the success of implant-supported oral rehabilitation is the marginal fit at prosthesis-implant interface [8–14]. Failures in casting of prosthesis can influence the fit on implants, leading to marginal and/or internal misfit, mechanical failures [15, 16], biological effects [9, 10, 13, 14, 17–21], and even loss of implant osseointegration.

There are several techniques for evaluation of the marginal fit between crown and implant. Clinically, it can be assessed with probes and radiographs. The* in vitro* evaluation can be done without destroying the samples using a stereomicroscope and, recently, computed microtomography (micro-CT) [22]. The measurement under a stereomicroscope is limited to two-dimensional (2D) view of the images. In contrast, the micro-CT allows measurement of misfit in both external and internal surfaces of the implant-supported assembly using three-dimensional (3D) images [22–24].

Since successful prosthetic treatments are related to appropriate marginal fit of dentures, the evaluation of marginal misfit at crown-implant interface is fundamental [25]. Therefore, the aim of this study was to evaluate, through computed microtomography, the internal and external marginal fit of single implant-supported metallic crowns fabricated by different methods. The null hypothesis assumed that the different manufacturing methods of the crowns would not influence the vertical and horizontal external marginal fit as well as internal fit to the implant.

## 2. Material and Methods

The variation in this in vitro study involved a manufacturing method of the single implant-supported crowns in four levels: conventional casting, induction casting, plasma casting, and CAD/CAM system. In total, four groups were tested (*n* = 8), including the following: GC, conventional casting with torch; GI, electromagnetic induction casting; GP, plasma casting; GCAD, machining by CAD/CAM system. The response variable was the internal and external marginal fit (*μ*m) of the crown/screw/implant assembly.

A total of 32 external hexagon implants (4.1 mm × 13.0 mm) (Pross; Dabi Atlante) attached to 32 castable UCLA abutments (Pross; Dabi Atlante) with regular platform of 4.1 mm in diameter were used in this study. The implants were embedded in the long axis using a dental surveyor (B2 Parallelometer; Bio-Art) in polyurethane-based homogeneous material (F-16 FastCast Polyurethane; Axson) into PVC tubes (Tigre S/A) with 25.0 mm in diameter × 20.0 mm in height.

For standardization of crowns waxing, a two-piece matrix was fabricated in condensation silicone (Zetaplus; Zhermack) based on waxing of an antirotational castable cylinder with 4.1 mm external hexagon platform (Pross; Dabi Atlante) using wax for metallic casting (Sculpture Wax PK; Kota Ind. e Com. Ltda.) and reproducing the anatomy of a maxillary canine. Then, waxing of the crowns was based on the two-piece matrix of condensation silicone, reproducing the anatomy. A total of 24 waxing processes were done and divided into 3 groups (GC, GI, and GP).

After waxing, the crowns were cast in Co-Cr alloy (Fit Cast Cobalto; Talmax) according to the 3 manufacturing methods of the groups (GC, GI, and GP). After casting, the crowns were harvested and sandblasted with 100 *μ*m aluminum oxide (Polidental Ind. e Com. Ltda.) under 80 psi (5,62 kgf/cm^2^) for removal of investment residues, and the casting tubes were cut with carborundum discs (Schelble). Finishing and polishing of the crowns were done with specific burs and pastes for metals (Exa-Cerapol; Edenta).

For fabrication of the crowns by scanning and machining in CAD/CAM system (GCAD), waxing was done as previously described for the other groups. For better accuracy and fit between crown and implant, the waxed crown was attached to the implant embedded in polyurethane and the assembly was positioned in the CAD/CAM system (Ceramill Map 300; Amann Girrbach). The crown was scanned, digitalized, and then designed in the system software (Ceramill Mind; Amann Girrbach), creating the 3D object shape. After getting the virtual model, the crowns were machined in Co-Cr alloy (Ceramill Sintron; Amann Girrbach). The finishing and polishing of the crowns (Exa-Cerapol; Edenta) were done according to the manufacturer's instructions.

**Figure 1:**
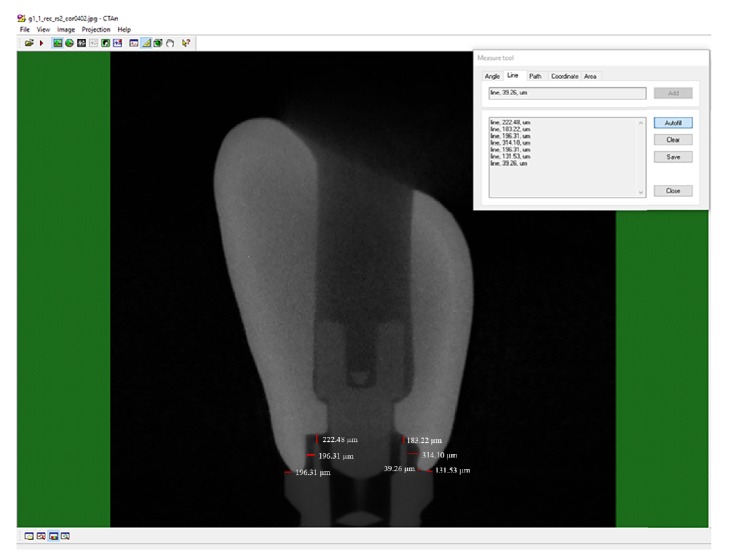
Marginal fit between the crown and implant for each sample, including external horizontal marginal fit, external vertical marginal fit, and internal marginal fit.

The internal and external marginal fit of the samples between the crown and implant were evaluated through micro-CT (Figure 1). The screw-retained crowns were tightened to their implants with torque recommended by the manufacturer (30 N·cm) using a digital torque gauge (TQ 680; Instrutherm Instrumentos de Medição Ltda.), with accuracy of 0.1 N·cm. Then, the samples were scanned in a microtomograph (Skyscan 1176; Bruker) attached to a microcomputer, using the following parameters: acceleration tension of 90 kV, current of 272 mA, 360° of rotation, resolution of 9 *μ*m, step of 0.5, frame 1, filter of 0.1 mm Cu. After scanning, the micro-CT images were reconstructed in the software NRecon (SkyScan; Bruker) with the following image adjustments: smoothing = 2; ring artifact correction = 10; beam hardening correction (%) = 70. Then, the images were transferred to the data viewer (SkyScan; Bruker) and reproduced in sagittal and coronal slices for evaluation of internal and external marginal fit in the CTAn (Skyscan; Bruker) using the software tool of linear measurement (Skyscan; Bruker).

For external marginal fit, both vertical and horizontal marginal fit were measured. The marginal fit was evaluated at sagittal and coronal planes using 3 slices for each sample: one central slice (C), corresponding to the implant center; one 0.5 mm slice below the central slice (C−); and one 0.5 mm slice above the central slice (C+). A total of 3 measures were done in each surface (buccal, palatal, mesial, and distal), totalizing 9 measures per surface and 36 measures per sample.

For internal fit, both vertical and horizontal internal fit were measured. The evaluation of internal fit (sum of vertical and horizontal internal fit) at both planes (sagittal and coronal) was done in the same slices selected for external marginal fit. In each slice, a total of 12 measures (6 vertical measures and 6 horizontal measures) were done, resulting in 108 measures per sample assuming analysis in 3 slices per plane.

The Kolmogorov–Smirnov statistical test for normality and Levéne test for homogeneity revealed normal distribution for data. Data of internal and external marginal fit was submitted to one-way ANOVA and Tukey post hoc test (*α* = .05). The analyses were performed in the software SPSS (IBM SPSS Statistics, v20.0; IBM Corp). The micro-CT was used for qualitative analysis of the images.

## 3. Results

Data of internal and external marginal misfit (mean and standard deviation) are shown in Table 1. The statistical analysis comparing the vertical marginal misfit of the crown/screw/implant assembly revealed no statistically significant difference between the groups (*P* = 0.721). For the horizontal marginal misfit of the crown/screw/implant assembly revealed no statistically significant difference between the groups GC, GI, and GP (*P* > 0.05). However, the groups GC, GI, and GP presented statistical difference (*P* ≤ 0.05) when compared to the group GCAD. Comparing the internal misfit of the crown/screw/implant assembly revealed no statistically significant difference between the groups GC, GI, and GP (*P* > 0.05). However, the groups GC, GI, and GP presented statistical difference (*P* ≤ 0.05) when compared to the group GCAD.

**Figure 2:**
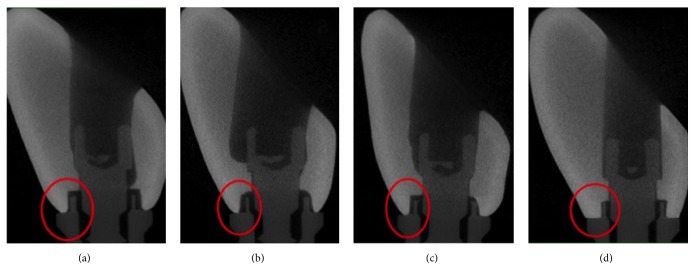
Sagittal section micro-CT image in relation to marginal fit between the crown and implant. (a) GC group; (b) GI group; (c) GP group; (d) GCAD group.

Qualitative analysis of the crown/screw/implant assembly revealed difference for both internal and external misfit between the groups (Figure 2). For the horizontal misfit in the groups GC, GI, and GP, underextended crowns were found as a consequence of lack of material. In the group GCAD, most of the samples showed images with appropriate crown/implant fit. However, some specimens presented overextension (material excess) of the crown in relation to the implant platform. For vertical misfit, the images showed intimate approximation between the crown surface and implant platform, revealing no vertical misfit in all groups. For internal misfit, the groups GC, GI, and GP presented images with excessive misfit between the crown and implant platform in all samples, in both vertical and horizontal directions. On the other hand, the group GCAD exhibited crowns with better fit when compared to the other groups, in both vertical and horizontal directions.

## 4. Discussion

The results of the present study rejected the null hypothesis that different manufacturing methods of crowns would not influence the horizontal marginal misfit and internal fit. However, data accepted the null hypothesis that the manufacturing methods would not influence the vertical marginal misfit.

The vertical marginal fit is assumed as a relevant feature of implant-supported prostheses as it provides stability and sealing of prosthesis-implant assembly under biofilm formation. Therefore, it preserves the local physiology and reduces the risk to peri-implantitis. Marginal gaps lead to proliferation of soft tissue and microorganisms [9, 10, 13, 14, 17–19], which results in biological problems and inflammation that can affect implants osseointegration [20].

In this study, satisfactory vertical marginal fit was found since the microtomographic images revealed intimate fit and no gap between crowns and implant platform in most of the samples, which enhances the biomechanical performance of the implant-supported crown. The measures of vertical marginal fit in this study (GC = 4.55 *μ*m; GI = 3.54 *μ*m; GP = 6.12 *μ*m; GCAD = 6.89 *μ*m) are in accordance with the minimum values of marginal misfit clinically acceptable (up to 10 *μ*m) found in the literature [26], concluding that all manufacturing methods of metallic crowns tested in this study are suitable. It is important to highlight that this feature can be related to the methodology of this study [13] when simulating a real clinical condition of 30 N·cm torque insertion in the retaining screw before misfit measurement as recommended by the manufacturer.

The evaluation of horizontal marginal misfit is also relevant to provide longevity of the implant-supported crowns. The horizontal misfit of crown in relation to the implant platform leads to bacterial proliferation and can affect the long- and medium-term treatment prognosis [8–14]. The implant-supported crowns must present extension (diameter) compatible with the implant diameter in order to provide appropriate biomechanical performance of the assembly. According to the literature, comparing both situations, overextended crowns are more prone to biofilm formation and consequent tissue inflammation and bone loss [8, 11, 13, 18, 21].

In the present study, the manufacturing method of the crowns has influenced the horizontal marginal fit to the implants. High values of horizontal misfit were found in the cast groups (GC = −189.35 *μ*m; GI = −196.43 *μ*m; GP = −206.34 *μ*m), assuming that negative values represent underextended crowns with lack of material. On the other hand, the group GCAD of CAD/CAM system was the best option since most of the crowns exhibited extension compatible with the implant diameter, with mean misfit value of 16.34 *μ*m.

Only few studies report numerical information about horizontal marginal misfit. Considering the qualitative analysis of images by micro-CT and numerical data, the values of horizontal marginal misfit in the cast groups are not acceptable because of substantial lack of material. This result can be explained by the previous waxing of the crowns before casting, which could result in dimensional change of the cervical diameter of castable UCLA abutment. In addition, since this region presents reduced amount of material, greater distortion of waxing might have occurred during investment heating for wax release. In order to minimize such effect, castable UCLA abutments with metallic base should be used because the metal does not change during casting as its melting point is higher than the temperature used for investment heating and compatible with the temperature for alloy injection [27], preserving the dimensions standardized during waxing.

In the group GCAD, with crowns machined by CAD/CAM system, digitalization provided samples with satisfactory accuracy of horizontal marginal fit within the limits previously determined. The results showed crowns with acceptable fit and better than the conditions found in the other groups (GC, GI, and GP), which is in agreement with previous studies [12, 28] that showed the advantages of CAD/CAM, especially in terms of the accuracy of the crowns.

However, as in all processing techniques, the CAD/CAM system is also prone to failure and has some disadvantages. In the present study, some samples of the group GCAD presented overextension of crown in relation to the implant platform. According to Davidowitz and Kotick [29] the misfit can be related to failures during fabrication of the prosthesis. Despite being a digital system, it only works after the command of an operator, which can cause failures during scanning, digitalization, and prosthesis design. Thus, the machining of prostheses using CAD/CAM system requires highly qualified professionals to enhance an appropriate final result.

The internal fit of crowns to implants should be also evaluated since it represents the adaptation of the prosthetic base to the implant platform. This situation provides stability to the assembly and avoids rotation, screw loosening, and fractures of screw and/or prosthesis [9, 10, 13, 14, 17, 18]. In the present study, the groups GC (157.87 *μ*m), GI (174.91 *μ*m), and GP (168.81 *μ*m) presented greater internal misfit than the group GCAD (68.54 *μ*m). This result can be explained by the limitations of each manufacturing method tested in the present study. The conventional casting method depends on operator skills for running the procedure. For induction casting, the appropriate control of temperature is essential for the final result of the prosthesis. In plasma casting, differences in pressure between the chambers and investment permeability and differences between the casting temperature and investment mold must be considered. Therefore, the features of each manufacturing method associated with the use of castable UCLA abutments in the cast groups might have affected the internal fit of crowns to the implant [3, 30]. On the other hand, in the group of CAD/CAM system, the use of a digital technology provided more accurate internal and horizontal marginal fit.

Considering the lack of studies about the influence of different manufacturing methods of implant-supported metallic crowns on the fit between prostheses and implants, this study showed that the CAD/CAM technique resulted in better adaptation compared to the casting methods evaluated even assuming its limitations. However, the results of an in vitro analysis must be carefully considered and transferred to clinical scenario since the performance of implant-supported prostheses depends on individual planning. Furthermore, it is important to highlight that the biomechanical interactions can be more complex and deeper than laboratorial tests.

## 5. Conclusions

Within the limitations of this in vitro study and according to the results, it may be concluded that the manufacturing methods of single metallic crowns did not affect the vertical marginal fit to the implant but have influenced the accuracy of internal and horizontal marginal fit, assuming that the crowns fabricated by CAD/CAM system presented the best results.

## Figures and Tables

**Table 1:** Mean misfit values in *μ*m and standard deviation in all groups for internal and external marginal fit.

Groups	External vertical marginal	External horizontal marginal	Internal misfit
GC	4.55 (4.36)^a^	−189.35 (17.57)^a^	157.87 (22.89)^a^
GI	3.54 (2.55)^a^	−196.43 (26.74)^a^	174.91 (19.00)^a^
GP	6.12 (8.78)^a^	−206.34 (24.02)^a^	168.81 (12.57)^a^
GCAD	6.89 (7.44)^a^	16.34 (29.32)^b^	68.6 (31.37)^b^

*Note.* Different lowercase letters in columns indicate statistically significant differences (*P* < 0.05).
